# Characterizing motivations for cannabis use in a cohort of people who use illicit drugs: A latent class analysis

**DOI:** 10.1371/journal.pone.0233463

**Published:** 2020-05-21

**Authors:** Stephanie Lake, Ekaterina Nosova, Jane Buxton, Zach Walsh, M. Eugenia Socías, Kanna Hayashi, Thomas Kerr, M. J. Milloy

**Affiliations:** 1 British Columbia Centre on Substance Use, Vancouver, BC, Canada; 2 School of Population and Public Health, University of British Columbia, Vancouver, BC, Canada; 3 British Columbia Centre for Disease Control, Vancouver, BC, Canada; 4 Department of Psychology, University of British Columbia, Kelowna, BC, Canada; 5 Department of Medicine, University of British Columbia, Vancouver, BC, Canada; 6 Faculty of Health Sciences, Simon Fraser University, Burnaby, BC, Canada; University of the West of Scotland, UNITED KINGDOM

## Abstract

**Background:**

Cannabis use is common among marginalized people who use illicit drugs (PWUD) but reasons for use remain poorly investigated. We sought to explore how different intentions for cannabis use relate to social, structural, and behavioural factors among PWUD in Vancouver, Canada.

**Methods:**

We used data from cannabis-using participants in two community-recruited prospective cohort studies of PWUD. Using latent class analysis, we identified discrete cannabis-using groups based on self-reported intentions for use. Generalized estimating equations were used to examine correlates of class membership.

**Results:**

Between June 2016 and December 2018, 2,686 observations from 897 participants cannabis-using PWUD were analyzed. Four latent classes of cannabis use emerged: Class 1 (31.6%), characterized by non-medical purposes; Class 2 (37.5%), characterized by non-pain therapeutic use (e.g., stress, nausea/loss of appetite, and insomnia); characterized by Class 3 (21.9%) predominantly pain relief; and Class 4 (9.0%), characterized by a wide range of therapeutic uses in addition to pain management, including insomnia, stress, nausea/loss of appetite, and harm reduction. Class-specific structural, substance-, and health-related differences were observed, including indicators of better physical and mental health among the “recreational” class, despite evidence of more structural vulnerabilities (e.g., homelessness, incarceration).

**Conclusions:**

Our findings demonstrate a wide spectrum of motivations for cannabis use among PWUD. We observed important health-related differences between latent classes, demonstrating possible unmet healthcare needs among PWUD reporting therapeutic cannabis use. These findings inform ongoing policy surrounding access to cannabis for harm reduction purposes and applications of medical cannabis for PWUD.

## Introduction

Cannabis is the most common illicit (i.e., internationally scheduled) drug consumed worldwide [1]. The preponderance of health and social research on cannabis tends to conceptualize its usage as non-medical (i.e., recreational) and often problematic in nature [2,3]. However, coinciding with policy reforms across the United States (US) and Canada, there has been a recent shift in the public perception of cannabis [4], bringing a growing interest in the range of its possible therapeutic applications. In Canada, more than 350,000 individuals possess a medical authorization to use cannabis for a range of conditions including chronic pain, insomnia, arthritis, and post-traumatic stress disorder [5,6].

Cannabis has long been incorporated into poly-substance use among marginalized people who use illicit drugs (PWUD). For example, approximately half of PWUD living with HIV in Vancouver, Canada report consuming cannabis in the previous six months [7], compared to a past-year prevalence of nearly 15% in the general population [8]. Despite this, the complex nature of cannabis use within the context of regular polysubstance use has been largely overlooked by the past two decades of epidemiological research involving PWUD. Emerging qualitative research has broached the idea that cannabis may serve a range of therapeutic purposes in these populations. PWUD describe purposefully engaging in cannabis use as a form of harm reduction (e.g., to manage opioid cravings or prevent escalation to higher-intensity opioid use [9,10]). These accounts are further supported by emerging experimental research demonstrating a potential role of cannabinoids in reducing opioid craving and withdrawal [11]. In light of an ongoing opioid overdose crisis throughout Canada and the United States in which marginalized PWUD have borne the brunt of morbidity and mortality, the evolving understanding of cannabis’ therapeutic potential raises important questions about whether—and, if so, how—cannabinoid-based interventions could be implemented and individual-tailored as a form of harm reduction [12].

Latent class analysis (LCA) is a statistical method that uses a combination of observed characteristics to identify discrete unobserved (i.e., latent) classes within a heterogeneous sample [13]. In recent years, a growing number of studies involving PWUD have employed LCA methodology to characterize poly-substance use and behavioural risk profiles [14–22]. Findings from these studies have highlighted important classifications of risk for overdose [19,22], HIV and hepatitis C virus transmission [16,18,20,21], injection-related infection and injury [14], sexual risk behaviours and sexually transmitted disease infection [15–17], and comorbid mental health concerns [20]. While some studies have employed LCA methodology to understand motivations for cannabis use [23,24], this research has tended to focus on young adult and student populations, conceptualizes cannabis use as inherently problematic, and leaves potential therapeutic motivations unexplored. For instance, a recent study of cannabis-using Americans aged 19–20 developed latent classes through examining motivations related to experimenting, getting high, relaxing, socializing, escaping problems/coping, peer pressure, dependence, and modifying the effects of other drugs in order to understand which classes were associated with problematic cannabis use 15 years later [24].

The objectives of the current study were to: 1) explore the range of therapeutic and non-therapeutic reasons for cannabis use among marginalized PWUD in Vancouver, Canada, during a community-wide opioid overdose crisis; 2) use LCA to assign membership to discrete groups of cannabis users based on reasons for use; and 3) estimate the relationships between class membership and a range of demographic, socio-structural, substance use, and other health-related factors.

## Materials and methods

### Study sample

Data for this study were derived from two ongoing open prospective cohort studies of PWUD in Vancouver, Canada: The Vancouver Injection Drug Users Study (VIDUS), consisting of HIV-negative people who inject drugs and the AIDS Care Cohort to evaluate Exposure to Survival Services (ACCESS) study, consisting of people living with HIV who use illicit drugs. Recruitment for both studies has been ongoing since 1996 (VIDUS) and 2005 (ACCESS) through extensive community-based outreach in various areas across Vancouver’s downtown core, including the Downtown Eastside (DTES), a low-income neighbourhood with an open illicit drug market and widespread marginalization and criminalization of PWUD. To be eligible for VIDUS, participants must report injecting drugs in the previous 30 days at enrolment. To be eligible for ACCESS, participants must report using an illicit drug (other than or in addition to non-medical cannabis, which was a controlled substance under Canadian law until October 17, 2018) in the previous 30 days at enrolment. For both cohorts, HIV serostatus is confirmed through serology. Other eligibility requirements include being aged 18 years or older, residing in the Greater Vancouver Regional District, and providing written informed consent. Aside from HIV-specific assessments, all study instruments and follow-up procedures are harmonized to facilitate combined data analysis and interpretation.

At study enrolment, participants complete an interviewer-administered baseline questionnaire. Every six months thereafter, participants complete a follow-up questionnaire. The questionnaires elicit information on socio-demographic characteristics, lifetime (baseline) and past six-month (baseline, follow-up) patterns of substance use, risk behaviours, health care utilization, social and structural exposures, and other health-related factors. Nurses collect blood samples for HIV testing (VIDUS) or HIV clinical monitoring (ACCESS) and hepatitis C virus serology, and provide referrals to appropriate health care services as needed. Participants are provided a $40 (CAD) honorarium for their participation at each study visit. Ethics approval for this study was granted by the University of British Columbia/Providence Health Care Research Ethics Board (VIDUS: H14-01396; ACCESS: H05-50233). Written informed consent was obtained from all study participants.

For the purposes of this study, the follow-up period was restricted to June 1, 2016 to December 1, 2018, as new cannabis measures—including information on reasons for use and sources of access—were added to the questionnaire in June 2016.

### Latent class model

#### Measures

All participants who self-reported any cannabis use in the previous six-month period were asked a follow-up question on the reason(s) why they used it. Participants were asked to endorse their reason(s) for cannabis use from a list of pre-determined categories (S1 Text) that emerged from a literature review and piloting process. Specifically, the categories were developed by cohort investigators and select study co-authors through knowledge of non-medical and medical uses of cannabis, with special attention to health issues that disproportionately affect PWUD (e.g., HIV and treatment side-effects, opioid withdrawal and craving, acute and chronic pain). The categories were distributed to study staff (including interviewers and research nurses with several years of experience working with the study population), peer research associates, community medical cannabis advocates, and healthcare providers, who provided additional input. There was also an option for participants to specify another reason under “Other” if it was missing from the option list. These string responses were scrutinized after each biannual interview round to identify any missing or emergent categories. Aside from “Other”, each of the categories was treated as a binary variable (yes vs. no).

#### Statistical analysis

First, the primary and senior author used a consensus-based approach to re-categorize all string responses under “Other” reasons for cannabis use into a pre-determined option wherever possible. A function heat map was generated to visualize clustering of individuals by reason(s) for cannabis use, and to inspect the number of responses for each variable. The variable “Help with HIV medications and AIDS symptoms” was removed at this stage due to low cell count; the two categories “Spiritual purposes” and “Creativity” were combined into a single variable for “Spirituality/Creativity”; and the two categories “To come down off of other drugs” and “To treat withdrawal” were combined into a single variable for “Manage addiction”.

Then, we conducted an LCA using the 13 reasons for use (S1 Text) to build empirically discrete groups based on the cannabis-using profiles of the cohort at each interview period. We used the R package *poLCA* to estimate the number of latent classes in the sample and the likelihood of each participant’s class membership. This software employs expectation-maximization and Newton-Raphson algorithms to find maximum likelihood estimates of the model parameters [25]. The classes were developed from observations at each interview period, meaning that individuals who contributed multiple observations to the data could belong to one class at one time and another class at another time over the study period. We tested a 2-, 3-, 4-, 5, and 6-class model using a combination of exploratory methods and *a priori* theoretical guidelines. Bayesian information criterion (BIC), Akaike information criterion (AIC), Pearson’s chi-square goodness of fit (χ^*2*^), and likelihood ratio (*G*^*2*^) statistics were examined for model fit. To avoid problems with generalizability that may arise from creating groups that are either very similar but extremely small or very large but extremely heterogeneous, we pre-specified that latent classes should represent no less than 5% and no more than 50% of sample observations. The number of times to estimate the model using different starting probability values was set to 20.

### Latent class regression

#### Measures

We considered socio-demographic, behavioural, and health-related factors hypothesized to vary by class membership. Unless otherwise specified, all variables are self-reported and refer to experiences in the six-month period prior to each study interview. Socio-demographic covariates included: age; sex; race (white vs. non-white); DTES residency; education level (≥high school vs. <high school); and legal employment. Socio-structural variables included: homelessness and incarceration. Substance use variables included: alcohol use; cocaine use; heroin injection (of note, during this study period, a high proportion of drugs sold as heroin in the community contain fentanyl [26], thus “heroin” injection refers to the injection of heroin as well as drugs sold as heroin); illicit prescription opioid use (i.e., non-medical use of prescribed, diverted, or counterfeit pharmaceutical opioids); crack use; cannabis use; and crystal methamphetamine use (all categorized as ≥ daily vs. < daily, to be consistent with previous analyses). Health-related variables included: hepatitis C serostatus at time of interview; HIV serostatus at time of interview; lifetime mental illness diagnosis; non-fatal overdose; pain severity in the past week (assessed with the Brief Pain Inventory, dichotomized into moderate-severe [mean score 4.5–10]) vs. none/mild [mean score 0–4.4]); depression, anxiety (each assessed with PROMIS short-form, dichotomized into moderate/severe [T-score ≥60] vs. none/mild [T-score ≤59.9]); self-perceived general health rating (good-excellent vs. poor-fair); and addiction treatment enrolment.

For descriptive purposes, we also considered the following sources for obtaining cannabis: dealer, friend, private grower (participant grows their own or pays someone else to grow it), compassion club (a cooperative providing low-cost cannabis to patients in financial need), retail dispensary (a municipally regulated or unregulated store selling products that are not legally regulated under the new federal law), a licensed medical cannabis producer (legal producers of medical cannabis selling to government-authorized patients only), or a legal store (legally regulated store for non-medical cannabis; added in the final interview period [June 1, 2018 –December 1, 2018], coinciding with legalization [October 17, 2018]).

#### Statistical analysis

First, we created binary outcome variables for each class (i.e., Class 1 vs. Other; Class 2 vs. Other, and so on). As the data could contain ≥1 observations from each participant, we used generalized estimating equations (GEE) to explore bivariable relationships between each variable above and class membership. This method estimates standard errors for each parameter using an exchangeable correlation structure to account for repeated measures within individuals [27]. Then, we built multivariable GEE models to predict membership in each class. These models included all covariates (aside from cannabis sources) that were associated with the outcome at *p*<0.10 in bivariable analyses. We used a backward selection approach starting by removing the covariate with the highest *p*-value and examining how model quasi-information criterion (QIC) changed with its removal. The final models were determined once QIC reached its lowest point. An odds ratio (OR) of < 1 indicates a negative association with the outcome (i.e., reduced odds of class membership for the exposure in question); whereas an OR of >1 indicates a positive association with the outcome (i.e., increased odds of class membership for the exposure in question).

All analyses were conducted in R (Version 3.5.0, R Foundation for Statistical Computing, Vienna, Austria). All *p*-values are two-sided.

## Results

### Sample characteristics

From June 2016 to December 2018, 1,447 PWUD completed 5,400 interviews (median number of interviews per participant = 3). Of these individuals, 897 (62.0%) reported using cannabis during 2686 (49.7%) study visits and were included in this study. Table 1 summarizes the socio-demographic characteristics of this sample at baseline. As shown, the median age of participants in this study was 47.7 years (Interquartile Range [IQR] = 38.4–54.6), one-third (33.3%) were women, and just under half (44.8%) were white. Over the study period, the median prevalence of past six-month ≥daily cannabis use ranged from 41.1% to 50.3% (median: 47.6%), whereas ≥ weekly use ranged from 23.2% to 29.9% (median: 27.7%) and < weekly use ranged from 21.1% to 27.1% (median: 23.9%).

**Table 1:** Baseline sociodemographic characteristics of 897 PWUD who reported cannabis use, June 2016 –December 2018.

Characteristic	N	%
Age (median, IQR)		
Median, IQR	47.7	38.4–54.6
Sex		
Male	598	66.7
Female	299	33.3
Race		
White	402	44.8
Visible minority	493	55.0
Education		
≥ High school	423	47.2
< High school	453	50.5
Legal employment^1^		
Yes	269	30.0
No	627	70.0
Homelessness^1^		
Yes	190	21.2
No	705	78.6
DTES residency^1^		
Yes	540	60.2
No	357	39.8
Incarceration^1^		
Yes	45	5.0
No	850	94.8

^1^Refers to exposures or experiences in the previous six months

### Selection of latent class model

We compared fit indices (AIC, BIC, χ^*2*^, *G*^*2*^) and class sizes of all five tested latent class models. The 2- and 3-class models were ruled out on the basis of poor model fit (S1 Table). A 6-class model was ruled out as two classes represented very small (<5%) portions of the data (S2 Table). The 5-class model also had one class with fewer than 5% of observation. As the 4- and 5-class model yielded similar fit statistics, the 4-class model was selected for superior interpretability.

### Latent classes

The representation of cannabis use motivations overall and across the emergent latent classes is summarized in Table 2. Of the 653 (72.8%) participants who completed more than one interview over the study period, 157 (24.0%) remained in the same class at each follow-up period. Of the remaining 496 participants who shifted classes during the study period, 353 (71.2%) occupied two classes at different points over the study period and 130 (26.2%) moved between three classes. A smaller number (n = 13, 2.6%) of respondents were categorized into each of the four classes at different times over the study period.

**Table 2:** Representation of cannabis use motivations overall and within latent classes among 897 PWUD who reported cannabis use, June 2016 –December 2018.

	Overall n = 2686;100%	Class 1:n = 848;31.6%	Class 2:n = 1007; 37.5%	Class 3:n = 588; 21.9%	Class 4: n = 243; 9.0%
**Cannabis use reasons**	Proportion of observations				
Intoxication	0.53	**1.00**	0.29	0.26	**0.50**
Pain relief	0.31	<0.01	<0.01	**1.00**	**1.00**
Mental health	0.08	0.02	0.10	0.06	0.21
Insomnia	0.32	0.00	**0.50**	0.22	**0.98**
Substitution	0.12	0.05	0.15	0.12	0.26
Nausea	0.29	0.00	0.45	0.27	**0.65**
Creativity / Spirituality	0.06	0.07	0.07	<0.01	0.12
Stress	0.32	0.13	0.45	0.17	**0.77**
Manage addiction	0.04	<0.01	0.06	0.02	0.09
**Characterization**	NA	Recreational	Non-pain therapeutic	Pain	Pain +

Class-specific proportions ≥0.50 are shown in bold.

### Class 1: “Recreational” class

Class 1, representing the second largest group (n observations = 848; 31.6%), was characterized as using cannabis predominantly for non-therapeutic (i.e., recreational) purposes such as intoxication, socialization, life enjoyment, etc. All members of this class indicated using cannabis for intoxication, and there was almost no therapeutic use of cannabis in this class aside from coping with stress, which was apparent in 13.4% of observations.

### Class 2: “Non-pain therapeutic” class

Class 2 represented the largest latent group (n observations = 1,007; 37.5%) and was distinct from other classes in the therapeutic use of cannabis for a number of conditions other than the management of pain. Specifically, a member of this class would have a substantial probability of using cannabis to treat insomnia (50%), nausea/loss of appetite (45%), and/or to manage stress (45%). As is the case with all classes, using cannabis for intoxication was also common (29% of observations).

### Class 3: “Pain” class

Class 3 (n observations = 588; 21.9%) was characterized as using cannabis predominantly to manage pain, as all members in this group used cannabis for pain relief. Although, there was some additional use of cannabis for other non-pain therapeutic reasons (including nausea/loss of appetite [26.9%], insomnia [21.9%], and stress [16.8%]), and recreational reasons (including intoxication [25.5%]), these therapeutic and recreational motives were under-represented compared to the below Class 4, in which pain tended to be addressed in conjunction with another health issue.

### Class 4: “Pain +” class

Class 4, the smallest group (n observations = 243; 9.0%), was distinct from other classes in that cannabis was serving at least one other therapeutic purpose (e.g., sleep [97.9%], nausea/loss of appetite [76.5%], stress [65.4%]) in addition to pain management (100%). Members of this class also engaged in recreational cannabis use more than the other two therapeutic classes (49.8%), and there was also a higher representation of less conventional, potentially therapeutic applications of cannabis compared to other classes. For example, some members classified into this group were also using cannabis to substitute for another substance including alcohol or opioids (26.3%), to manage a mental illness (21.4%), for spiritual purposes (12.4%), or to treat addiction or manage withdrawal (9.5%).

### Sources of cannabis across classes

**Fig 1:**
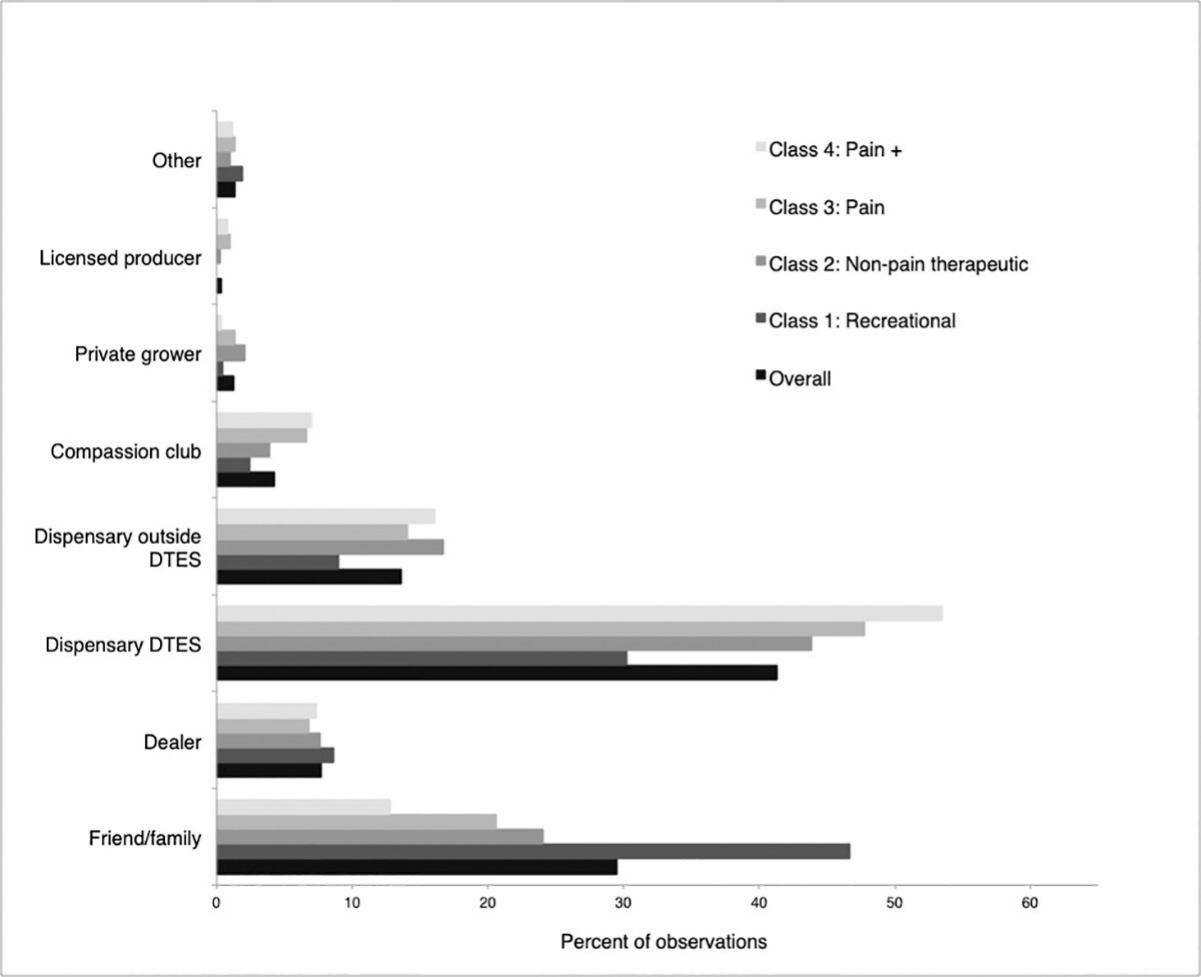
Primary source of cannabis reported overall and by class membership, June 2016 –December 2018 (n = 897, observations = 2686).

Illicit dispensaries in the DTES neighbourhood were the most common source of cannabis overall, reported in over 50% of interview periods. Many individuals also reported acquiring cannabis from a friend/family member (34.9% of observations) or a dispensary outside of the DTES (16.3% of observations). Less common sources were dealers, a compassion club, private growers, and licensed producers. As shown in Fig 1, dispensaries (mostly those located in the DTES neighbourhood) were the “most important” source of cannabis for the majority of members from classes 2 (60.6%), 3 (61.9%), and 4 (69.5%), while friends/family was the most commonly reported primary source of cannabis in class 1 (46.7%). Bivariable analyses confirmed these class differences in cannabis access patterns (Table 3). Additionally, membership in class 3 (“Pain”) was positively associated with obtaining cannabis from a compassion club. There were very few reports of accessing cannabis through the authorized medical cannabis system (n = 12; 0.45%), and only 2 participants (from Classes 3 and 4; 0.07%) accessed legal non-medical cannabis after legalization.

**Table 3:** Bivariable associations between cannabis sources and cannabis use classes.

Cannabis source	Class 1:“Recreational”		Class 2:“Non-pain therapeutic”		Class 3:“Pain”		Class 4: “Pain +”	
	Odds Ratio(95% CI)	*p*-value	Odds Ratio(95% CI)	*p*-value	Odds Ratio(95% CI)	*p*-value	Odds Ratio(95% CI)	*p*-value
Friend or private grower	**1.97 (1.64–2.36)**	**<0.001**	**0.74 (0.62–0.89)**	**0.001**	**0.72 (0.58–0.87)**	**0.001**	**0.59 (0.43–0.81)**	**0.001**
Dispensary	**0.40 (0.33–0.48)**	**<0.001**	**1.54(1.29–1.82)**	**<0.001**	**1.45 (1.17–1.80)**	**0.001**	**2.03 (1.46–2.83)**	**<0.001**
Dealer	0.99 (0.78–1.26)	0.917	1.03 (0.81–1.31)	0.814	0.91 (0.67–1.23)	0.527	1.08 (0.71–1.65)	0.724
Compassion club	**0.74 (0.55–0.99)**	**0.044**	0.83 (0.59–1.16)	0.268	**1.48 (1.02–2.14)**	**0.039**	1.54 (0.92–2.55)	0.098
Medical cannabis licensed producer	(NA)	(NA)	0.65 (0.21–1.99)	0.455	2.26 (0.70–7.27)	0.172	3.33 (0.83–13.41)	0.091

95% CI = 95% Confidence Interval; *p<0.05; **p<0.001; NA = 0 cells counts for medical cannabis licensed producer in class 1; Bold indicates statistical significance at *p*<0.05

### Latent class analysis using GEE

Class 1 (“Recreational”) was significantly associated with a host of socio-demographic factors at the bivariable-level, including being male, living in the DTES, and experiencing recent homelessness and/or incarceration. The odds of daily alcohol use was increased in this class, while the odds of daily prescription opioid and daily cannabis use were significantly lower than other classes. Members of this class had lower odds of reporting common co-morbidities experienced among PWUD. Specifically, they were less likely to be living with HIV, have a mental illness diagnosis, or live with moderate-severe levels of pain. They were also more likely to report good-to-excellent self-perceived health (Table 4). In a multivariable model, the associations with DTES residency, homelessness, incarceration, daily prescription opioid use, and self-perceived health were removed from consideration or lost significance (Table 5).

**Table 4:** Bivariable generalized estimating equations of factors associated with membership in each latent class (n = 897, observations = 2686).

Characteristics	Class 1:“Recreational”		Class 2:“Non-pain therapeutic”		Class 3:“Pain”		Class 4:“Pain +”	
	Odds Ratio(95% CI)	*p*-value	Odds Ratio(95% CI)	*p*-value	Odds Ratio(95% CI)	*p*-value	Odds Ratio(95% CI)	*p*-value
**Socio-demographic factors**								
Age	0.99 (0.98–1.00)	0.086	1.00 (0.99–1.01)	0.777	**1.01 (1.00–1.02)**	**0.038**	1.00 (0.98–1.01)	0.719
Male	**1.36 (1.08–1.71)**	**0.010**	1.08 (0.88–1.33)	0.471	**0.62 (0.48–0.80)**	**<0.001**	0.95 (0.69–1.29)	0.726
White	0.88 (0.71–1.09)	0.229	1.07 (0.88–1.31)	0.487	0.96 (0.75–1.23)	0.763	1.23 (0.91–1.66)	0.179
≥ High school education	1.22 (0.98–1.51)	0.076	0.84 (0.69–1.03)	0.089	1.01 (0.79–1.29)	0.928	0.92 (0.68–1.24)	0.582
Legal employment	1.04 (0.88–1.24)	0.624	0.93 (0.78–1.11)	0.423	0.99 (0.81–1.22)	0.943	1.03 (0.76–1.40)	0.858
DTES residency^1^	**1.22 (1.00–1.48)**	**0.049**	0.89 (0.74–1.06)	0.197	0.87(0.69–1.09)	0.225	1.21 (0.89–1.64)	0.219
Homelessness^1^	**1.26 (1.01–1.59)**	**0.042**	0.94 (0.74–1.18)	0.588	**0.74 (0.56–0.99)**	**0.044**	1.13 (0.77–1.67)	0.531
Incarceration^1^	**1.53 (1.03–2.27)**	**0.033**	0.69 (0.45–1.06)	0.093	0.69 (0.44–1.09)	0.114	1.42 (0.80–2.52)	0.225
**Substance use factors**								
≥ Daily alcohol use^1^	**1.59 (1.23–2.06)**	**<0.001**	0.87 (0.66–1.16)	0.348	0.78 (0.55–1.10)	0.156	0.62 (0.37–1.05)	0.075
≥ Daily cocaine use^1^	1.18 (0.77–1.80)	0.444	0.84 (0.57–1.23)	0.365	0.57 (0.30–1.08)	0.084	**1.92 (1.13–3.26)**	**0.016**
≥ Daily heroin injection^1^	1.20 (0.95–1.51)	0.126	0.94 (0.75–1.18)	0.600	0.78 (0.60–1.02)	0.074	1.11 (0.78–1.59)	0.556
≥ Daily PO use^1^	**0.67 (0.47–0.97)**	**0.032**	0.89 (0.59–1.34)	0.576	**1.72 (1.07–2.77)**	**0.024**	1.21 (0.58–2.55)	0.609
≥ Daily crack use^1^	1.01 (0.73–1.42)	0.931	1.05 (0.77–1.44)	0.765	0.85 (0.58–1.24)	0.397	1.09 (0.66–1.81)	0.741
≥ Daily cannabis use^1^	**0.35 (0.29–0.42)**	**<0.001**	**1.20 (1.01–1.43)**	**0.039**	**1.54 (1.25–1.89)**	**<0.001**	**4.61 (3.30–6.44)**	**<0.001**
≥ Daily crystal meth use^1^	0.87 (0.69–1.11)	0.262	1.07 (0.85–1.35)	0.553	1.02 (0.78–1.33)	0.895	1.05 (0.70–1.57)	0.826
**Health-related factors**								
Hepatitis C +	1.02 (0.78–1.33)	0.895	0.94 (0.74–1.21)	0.647	1.21 (0.88–1.67)	0.249	0.81 (0.55–1.20)	0.295
HIV +	**0.58 (0.47–0.73)**	**<0.001**	**1.59 (1.31–1.94)**	**<0.001**	1.11 (0.86–1.42)	0.429	0.92 (0.67–1.25)	0.579
Mental illness diagnosis	**0.64 (0.51–0.79)**	**<0.001**	1.05 (0.86–1.29)	0.634	**1.59 (1.21–2.09)**	**0.001**	1.16 (0.84–1.61)	0.355
Non-fatal overdose^1^	1.09 (0.86–1.39)	0.462	1.19 (0.95–1.50)	0.125	**0.71 (0.54–0.94)**	**0.015**	0.77 (0.50–1.19)	0.245
Moderate-severe pain^2^	**0.57 (0.48–0.68)**	**<0.001**	**0.69 (0.59–0.82)**	**<0.001**	**2.95 (2.41–3.60)**	**<0.001**	1.26 (0.94–1.69)	0.125
Moderate-severe depression^2^	1.05 (0.82–1.34)	0.713	0.83 (0.66–1.05)	0.127	1.05 (0.80–1.39)	0.707	1.38 (0.96–2.00)	0.084
Moderate-severe anxiety^2^	0.83 (0.67–1.03)	0.096	0.99 (0.80–1.23)	0.930	0.94 (0.74–1.21)	0.648	**1.75 (1.25–2.44)**	**0.001**
Good-excellent perceived health	**1.31 (1.10–1.56)**	**0.002**	1.05 (0.87–1.25)	0.632	**0.70 (0.57–0.86)**	**0.001**	0.94 (0.70–1.26)	0.674
Addiction treatment^1^	1.07 (0.90–1.28)	0.440	**0.78 (0.65–0.93)**	**0.006**	1.18 (0.94–1.48)	0.145	1.27 (0.93–1.73)	0.136

95% CI = 95% Confidence Interval; IQR = Interquartile Range; PO = Pharmaceutical opioid

^1^Past six months

^2^Past week; Bold indicates statistical significance at *p*<0.05

**Table 5:** Multivariable generalized estimating equations of factors independently associated with membership in each latent class (n = 897, observations = 2686).

Characteristics	Class 1:“Recreational”		Class 2:“Non-pain therapeutic”		Class 3:“Pain”		Class 4:“Pain +”	
	Odds Ratio(95% CI)	*p*-value	Odds Ratio(95% CI)	*p*-value	Odds Ratio(95% CI)	*p*-value	Odds Ratio(95% CI)	*p*-value
**Socio-demographic factors**								
Age	0.99(0.98–1.00)	0.156	--	--	--	--	--	--
Male sex	**1.62(1.23–2.13)**	**0.001**	--	--	**0.65(0.51–0.84)**	**0.001**	--	--
≥ High school education	--	--	0.86(0.71–1.05)	0.147	--	--	--	--
Homelessness^1^	1.34(0.97–1.83)	0.074	--	--	--	--	--	--
Incarceration^1^	--	--	0.71(0.46–1.10)	0.127	--	--	--	--
**Substance use factors**								
≥ Daily alcohol use^1^	**1.77(1.26–2.48)**	**0.001**	--	--	--	--	**0.44(0.23–0.82)**	**0.010**
≥ Daily cocaine use^1^	--	--	--	--	--	--	**2.07(1.11–3.85)**	**0.021**
≥ Daily heroin injection^1^	--	--	--	--	**0.74(0.55–0.98)**	**0.039**	--	--
≥ Daily cannabis use^1^	**0.27(0.21–0.34)**	**<0.001**	1.15(0.96–1.38)	0.124	**1.58(1.28–1.97)**	**<0.001**	**5.39(3.68–7.91)**	**<0.001**
**Health-related factors**								
HIV +	**0.59(0.45–0.77)**	**<0.001**	**1.57(1.28–1.92)**	**<0.001**	--	--	--	--
Mental illness diagnosis	**0.72(0.56–0.93)**	**0.013**	--	--	**1.39(1.07–1.82)**	**0.015**	--	--
Non-fatal overdose^1^	--	--	--	--	**0.66(0.49–0.89)**	**0.007**	--	--
Moderate-severe pain^2^	**0.52(0.41–0.67)**	**<0.001**	**0.70(0.58–0.83)**	**<0.001**	**2.76(2.24–3.40)**	**<0.001**	--	--
Moderate-severe anxiety^2^	0.79(0.61–1.02)	0.073	--	--	--	--	**1.93(1.37–2.72)**	**<0.001**
Good-excellent perceived health	1.21(0.96–1.52)	0.106	--	--	0.81(0.65–1.00)	0.051	--	--
Addiction treatment^1^	--	--	**0.83** **(0.69–1.00)**	**0.049**	--	--	--	--

95% CI = 95% Confidence Interval; IQR = Interquartile Range; PO = Pharmaceutical opioid

^1^Past six months

^2^Past week; Bold indicates statistical significance at *p*<0.05; --indicates variable was not included in the final multivariable model

As shown in Table 4, membership in class 2 (“Non-pain therapeutic”) was significantly and positively associated with using cannabis daily and living with HIV, and negatively associated with moderate-severe pain and being enrolled in addiction treatment. In a multivariable model, all associations remained significant except daily cannabis use (Table 5).

At the bivariable-level (Table 4), members of class 3 (“Pain”) were slightly older than members of other classes, and less likely to be male or experiencing homelessness. Membership in this class was also positively associated with daily cannabis use and daily prescription opioid use. In terms of health-related factors, this group had significantly increased odds of a lifetime mental illness diagnosis and experiencing moderate-severe pain, and significantly lower odds of good-excellent self-perceived quality of health; however, they also had significantly lower odds of experiencing a recent non-fatal overdose. In a multivariable model, age, homelessness, and daily prescription opioid use were removed from consideration, and an additional negative association with daily heroin injection emerged (*p* = 0.04; Table 5).

Membership in class 4 (“Pain +”) was not associated with any socio-demographic characteristics. In terms of substance use factors, members of this class had increased odds of daily cocaine and cannabis use. They also had increased odds of experiencing moderate-severe levels of anxiety. These associations remained significant in a multivariable model, along with an emergent negative association with daily alcohol use (Table 5). Despite the high prevalence of cannabis use for pain management, the odds of moderate-severe pain were not significantly increased for this class relative to the others (bivariable *p* = 0.125).

## Discussion

We used latent class analysis to categorize 897 PWUD who use cannabis into groups defined by their therapeutic and non-therapeutic motivations for cannabis use. Three classes (2–4) encompassing over two-thirds (n = 1,838; 68.4%) of the sample observations were characterized—in full or in part—by some type of therapeutic cannabis use, and all four classes included individuals who also used cannabis for intoxication (class-specific prevalence: 25–100%), demonstrating substantial overlap in therapeutic and non-therapeutic use. This finding is consistent with recent survey data from medical cannabis and general population samples in Canada [28] and the United States showing a high prevalence of engaging in both medical and recreational cannabis use, especially in jurisdictions where non-medical cannabis is also legal [29,30].

Bivariable and multivariable analyses of class membership revealed several notable differences. First, daily cannabis use was significantly and positively associated with all three of the classes that endorsed therapeutic cannabis use (classes 2–4), and negatively associated with the class characterized by non-medical cannabis use (class 1). This trend, coupled with the increased likelihood of accessing cannabis through a more consistent and reliable source such as a dispensary (classes 2–4), compassion club (class 3), or the licensed medical cannabis system (class 4), and not through informal/illicit sources (e.g., friend/family, private grower, or dealer), suggests an intentional incorporation of cannabis into a daily routine among therapeutic users. Although daily use is often considered a component of problematic cannabis use (e.g., using the WHO ASSIST [31]), medical users tend to exhibit lower scores on cannabis use problems components of such screening/diagnostic tools, despite a higher likelihood of daily use [32,33]. In contrast to the therapeutic groups, our data suggests that cannabis use within the non-therapeutic class may reflect opportunistic cannabis use as part of a broader pattern of poly-substance use.

Second, despite increased likelihoods of social and structural vulnerabilities (e.g., homelessness, incarceration) in the recreational use class, several positive health outcomes (e.g., better self-perceived general health, less pain, less anxiety, lack of diagnosed mental illness, HIV-negative) were associated with membership in the class, while members of the classes characterized by therapeutic use tended to exhibit poorer indicators of health. These patterns are likely indicative of frequent cannabis use to address poor health rather than poor health resulting from frequent cannabis use, as has been described previously [34,35]. Our findings suggest that daily cannabis use among PWUD may signify an unmet healthcare need, such as under- or unmanaged chronic pain or mental illness. These correlations also point to the need to conduct clinical studies to better understand the independent effects of cannabis use on health and well-being, especially among marginalized PWUD.

Our community and many others across Canada and the United States are experiencing an opioid overdose crisis rooted, in part, in inadequately or inappropriately-managed chronic pain [36,37] and sparked by widespread exposure to an unregulated illicit opioid supply contaminated with potent opioid analogues [38]. It is notable that members of the pain relief class had lower odds of reporting a recent non-fatal overdose and daily heroin injection relative to the other classes. Previous analyses of state-level data from the United States have described reduced rates of opioid overdose linked to cannabis legalization [39–41] thought to emerge from individuals replacing opioids with cannabis for pain relief [42–44], but a more recent study presents updated population-level data to dispute this hypothesis [45], highlighting a clear need for individual-level research. This study is the first, to our knowledge, to observe a lower likelihood of accidental overdose among high-risk PWUD using cannabis for pain. Although it is possible that cannabis is being used in place of (drugs sold as) heroin to manage pain within this class, of note is the bivariable association with daily prescription opioid use (Table 4). The negative association with overdose observed here could be partially explained by the use of regulated prescription opioids over unregulated and increasingly toxic opioids to manage pain [38,46]. This variable, however, represents all non-medical PO use—including diverted or not-as-prescribed use of pharmaceutically regulated POs as well as unregulated counterfeit pills. Our finding may also reflect an opioid-sparing effect of cannabis, whereby opioids are not replaced, but the dosage or frequency of opioid required for analgesia is reduced with the use of cannabis [47]. Cooper *et al*. recently tested this phenomenon in a blinded, placebo-controlled experimental study among 18 healthy adults, demonstrating significantly reduced pain responses from a sub-therapeutic dose (2.5 mg) of oxycodone when co-administered with smoked cannabis (5.6% THC) [48]. Future research is needed to investigate exposure to opioids—including heroin, fentanyl, and other opioid analgesics—among PWUD with pain, including longitudinal studies to test the opioid-sparing hypothesis.

Finally, although class 4 (“Pain +”) contained a higher proportion of observations in which cannabis was used as a strategy to reduce other high-risk substance use and manage symptoms of addiction, we did not observe significantly reduced odds of daily use of opioids, crack, or methamphetamine in this group. We did, however, note that daily alcohol use was less likely in this class. Previous research involving frequent users of crack-cocaine in this population demonstrates the intentional use of cannabis as a strategy to reduce frequency of crack use [49]. Interestingly, engaging in daily use of cocaine was positively associated with class 4 membership, possibly reflecting the strategy of using cannabis to “come down” from or stabilize the effects of cocaine [50,51], including to facilitate sleep [51]. Although there was a high proportion of observations in which cannabis was reportedly used for insomnia (98%), the lack of association with high frequency use of other stimulants in this class suggests further investigation is needed.

Notably, we observed very few reports of individuals accessing cannabis through legal routes—either the medical cannabis system (existing in various forms since 2001) or the new market for legal non-medical cannabis established in October 2018. The low levels of legal medical cannabis use might be a product of barriers to access that have been previous reported in other populations, including high prices, lack of consistent product supply and difficulties acquiring authorizations from physicians [52]. As our study period only included the first six weeks following non-medical cannabis legalization, we hesitate to draw any conclusions from the lack of reports of accessing that market, and we note that only online legal sales were available during that period. Illegal retail dispensaries were the most common source of cannabis and more likely to be accessed during membership in a therapeutic class, highlighting some possible negative consequences vulnerable PWUD may face as a result of restrictive approaches to cannabis legalization. Specifically, a financial barrier to the legal market is likely to arise when these illegal dispensaries are forced to closed—an intention the federal, provincial and municipal authorities have affirmed in planning implementation of the regulatory system for legal cannabis [53,54]. Future research should monitor the possible unintended health and social impacts of eliminating these low-barrier sources of cannabis, including uptake of illicit opioids for pain relief.

The findings of this study should be interpreted in the context of several limitations. First, generalizability to the local PWUD population and to other groups of PWUD may not be warranted, and special attention should be paid to the older age (i.e., potential survivorship bias) and high representation of HIV-positive respondents through the amalgamation of ACCESS data. All data other than HIV and hepatitis C serostatus are based on self-report. However, the likelihood of responding according to social norms is minimized as self-report of illicit drug use is already an eligibility requirement to be interviewed for these studies. Furthermore, previous research supports that PWUD provide accurate and reliable accounts of their recent drug use history [55]. Third, our data did not capture the type (e.g., cultivar, cannabinoid concentration) or quantity (e.g., grams) of cannabis used nor the mode(s) used to consume it. We also did not screen for cannabis use disorder or possible cannabis-related harms during the study period. These details would provide additional context to the therapeutic and non-therapeutic cannabis use profiles among PWUD and should be examined in future research. Fourth, reasons for cannabis use outside of those defined in the study questionnaire required re-categorization during data analysis in order to be considered for the latent class analysis. While most string responses mapped readily to a pre-determined category (e.g., “cut back on cigarettes” = Substitution), others were less clear (e.g., “menopause”, “helps me function”) and are subject to misclassification error. However, these responses accounted for <3% of all observations and are unlikely to have meaningfully impacted our findings. Fifth, this analysis is based on the results of repeated surveys and although we accounted for within-person observations over time, temporality and causality cannot be inferred from this analysis. Finally, although the final six weeks of the approximately 130-week study period occurred after Canada legalized non-medical cannabis, we do not believe this regulatory change substantially influenced our study findings. Personal possession and use of cannabis has long been decriminalized in Vancouver; no retail outlets selling legal cannabis existed in Vancouver during the study period.

## Conclusions

In this study of PWUD contending with numerous social and structural vulnerabilities and experiencing high rates of drug-related harms, we found that motivations for cannabis use occur on a spectrum from specific therapeutic (e.g., direct pain management) to broader non-medical use, with a high degree of overlap in between. Our findings suggest that an individual’s intentions around cannabis use may be closely linked to social and environmental vulnerabilities, co-occurring substance use, and states of physical and mental health. Certain indicators of poor physical and mental health were more likely among classes defined by at least some therapeutic use, suggesting that engaging in cannabis use for therapeutic purposes might signify an otherwise unmet healthcare need. Health care professionals working with marginalized PWUD should invite open conversations about cannabis use and intentions with patients to determine how medical cannabis might fit into a comprehensive treatment plan, or if a more suitable treatment is available—particularly in the context of health conditions tightly linked to long-term use of illicit drugs (e.g., pain, nausea/loss of appetite, insomnia, HIV symptoms). Although Canada has recently legalized non-medical cannabis, we found almost no reports of PWUD accessing cannabis via legal non-medical or medical cannabis systems. This finding highlights possible barriers to access among a population who may benefit from regulated products and who risk being further criminalized for their participation in the unregulated cannabis market.

## Supporting information

S1 Text Cannabis use reasons. (DOCX)

S1 Table Fit statistics for latent class models fit to 2686 observations from 897 PWUD. (DOCX)

S2 Table Number (%) of observations in each class of latent classes fit to 2686 observations from 897 PWUD. (DOCX)
