# Supplementary material for: Characterizing motivations for cannabis use in a cohort of people who use illicit drugs: A latent class analysis
Source: PLoS One. 2020 May 21;15(5):e0233463. doi: 10.1371/journal.pone.0233463 (PMC7241718; doi:10.1371/journal.pone.0233463)
Supplement: S1 Text — (DOCX) [file pone.0233463.s001.docx]

**S1 Text.** Cannabis use reasons

| **(1)** To relieve pain, including multiple sclerosis (MS), arthritis, etc. |
| --- |
| **(2)** To help with sleep |
| **(3)** To help with HIV medications and AIDS symptoms |
| **(4)** To treat nausea or loss of appetite |
| **(5)** To substitute for other substances including heroin, crack, meth, or alcohol |
| **(6)** To relieve stress |
| **(7)** To treat a mental health concern other than addiction |
| **(9)** For spiritual purposes |
| **(10)** For creativity  **(11)** To get high, recreation, socialize |
| **(12)** To come down off of other drugs |
| **(13)** To treat withdrawal |
