# Supplementary material for: Characterizing motivations for cannabis use in a cohort of people who use illicit drugs: A latent class analysis
Source: PLoS One. 2020 May 21;15(5):e0233463. doi: 10.1371/journal.pone.0233463 (PMC7241718; doi:10.1371/journal.pone.0233463)
Supplement: S1 Table — (DOCX) [file pone.0233463.s002.docx]

**S1 Table.** Fit statistics for latent class models fit to 2686 observations from 897 PWUD

| **Number of classes** | **AIC** | **BIC** | **χ*^2^*** | ***G^2^*** |
| --- | --- | --- | --- | --- |
| 2 | 21578.24 | 21690.26 | 18346.24 | 913.85 |
| 3 | 21468.68 | 21639.66 | 12419.91 | 784.29 |
| 4 | 21382.55 | 21612.48 | 1786.88 | 678.16 |
| 5 | 21308.49 | **21597.39** | 903.14 | 584.1 |
| 6 | **21262.08** | 21609.93 | **877.21** | **517.69** |
| Bold = Ideal class model based on fit statistic; Shaded = Class model selected  AIC = Akaike information criterion; BIC = Bayesian information criterion; χ*^2^ =* Pearson’s chi-square goodness of fit; *G^2^* = Likelihood ratio / deviance statistic | | | | |
