# Supplementary material for: Characterizing motivations for cannabis use in a cohort of people who use illicit drugs: A latent class analysis
Source: PLoS One. 2020 May 21;15(5):e0233463. doi: 10.1371/journal.pone.0233463 (PMC7241718; doi:10.1371/journal.pone.0233463)
Supplement: S2 Table — (DOCX) [file pone.0233463.s003.docx]

**S2 Table.** Number (%) of observations in each class of latent classes fit to 2686 observations from 897 PWUD

| **Number of classes** | **Class 1** | **Class 2** | **Class 3** | **Class 4** | **Class 5** | **Class 6** |
| --- | --- | --- | --- | --- | --- | --- |
| 2 | 845 (31.5) | **1841 (68.5)** | - | - | - | - |
| 3 | 179 (6.7) | 909 (33.8) | 1598 (59.5) | - | - | - |
| 4 | 1007 (37.5) | 588 (21.9) | 848 (31.6) | 243 (9.0) | - | - |
| 5 | **124 (4.6)** | 961 (35.8) | 350 (13.0) | 874 (32.5) | 377 (14.0) | - |
| 6 | 1031 (38.4) | **117 (4.4)** | 780 (29.0) | **130 (4.8)** | 284 (10.6) | 344 (12.8) |
| Bold = Class is considered to have low interpretability based on number of observations; Shaded = Class model selected | | | | | | |
